# Effect of adaptive laboratory evolution of engineered *Escherichia coli* in acetate on the biosynthesis of succinic acid from glucose in two-stage cultivation

**DOI:** 10.1186/s40643-024-00749-5

**Published:** 2024-04-05

**Authors:** Jiaping Jiang, Yuanchan Luo, Peng Fei, Zhengtong Zhu, Jing Peng, Juefeng Lu, Du Zhu, Hui Wu

**Affiliations:** 1 State Key Laboratory of Bioreactor Engineering, Shanghai Frontiers Science Center of Optogenetic Techniques for Cell Metabolism, School of Biotechnology, East China University of Science and Technology, 130 Meilong Road, Shanghai, 200237 China; 2 MOE Key Laboratory of Bio-Intelligent Manufacturing, School of Bioengineering, Dalian University of Technology, Dalian, China; 3 Shanghai Collaborative Innovation Center for Biomanufacturing Technology, 130 Meilong Road, Shanghai, 200237 China; 4 Key Lab of Bioprocess Engineering of Jiangxi Province, College of Life Sciences, Jiangxi Science and Technology Normal University, Nanchang, 330013 China

**Keywords:** Adaptive laboratory evolution, *E. coli*, Acetate, Succinic acid, Two-stage fermentation

## Abstract

*Escherichia coli* MLB (MG1655 Δ*pflB* Δ*ldhA*), which can hardly grow on glucose with little succinate accumulation under anaerobic conditions. Two-stage fermentation is a fermentation in which the first stage is used for cell growth and the second stage is used for product production. The ability of glucose consumption and succinate production of MLB under anaerobic conditions can be improved significantly by using acetate as the solo carbon source under aerobic condition during the two-stage fermentation. Then, the adaptive laboratory evolution (ALE) of growing on acetate was applied here. We assumed that the activities of succinate production related enzymes might be further improved in this study. *E. coli* MLB46-05 evolved from MLB and it had an improved growth phenotype on acetate. Interestingly, in MLB46-05, the yield and tolerance of succinic acid in the anaerobic condition of two-stage fermentation were improved significantly. According to transcriptome analysis, upregulation of the glyoxylate cycle and the activity of stress regulatory factors are the possible reasons for the elevated yield. And the increased tolerance to acetate made it more tolerant to high concentrations of glucose and succinate. Finally, strain MLB46-05 produced 111 g/L of succinic acid with a product yield of 0.74 g/g glucose.

**Synopsis:**

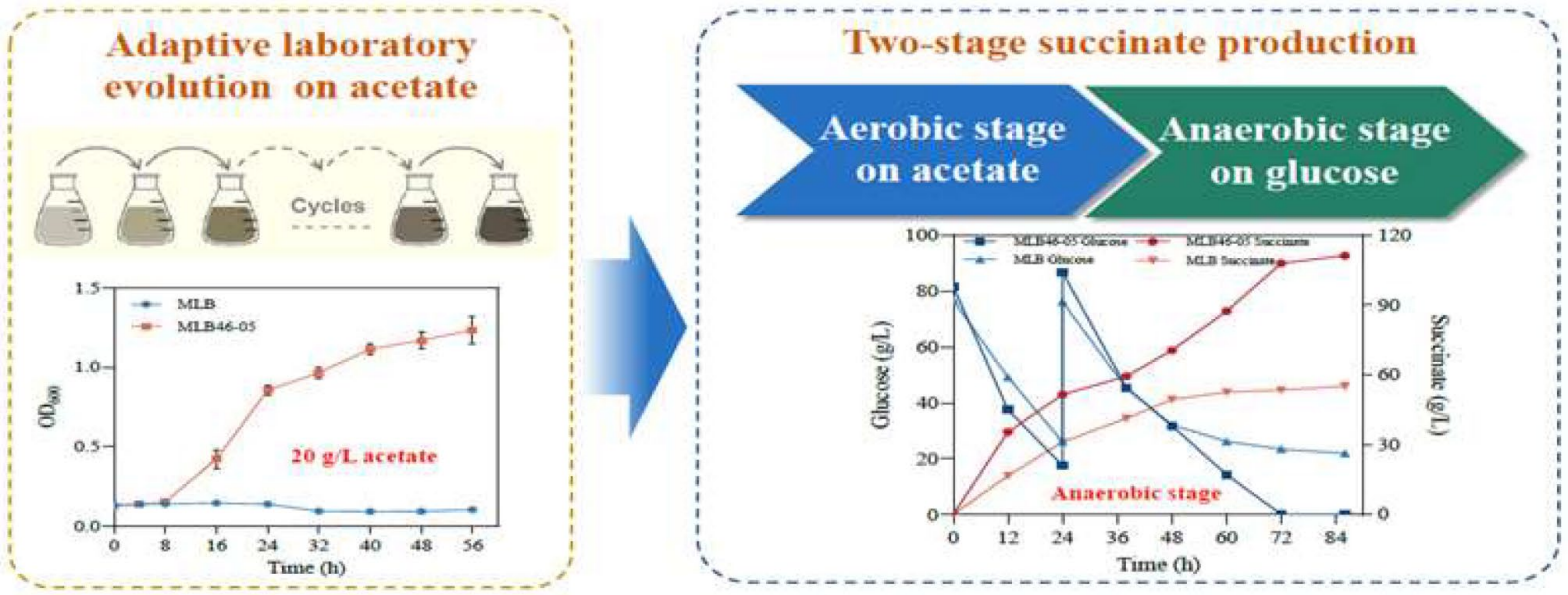

**Supplementary Information:**

The online version contains supplementary material available at 10.1186/s40643-024-00749-5.

## Introduction

Succinic acid is a high-value platform compound and it was selected as one of the most important bio-based chemicals back in 2004 and 2010 (Choi et al. 2015; Wu et al. 2007). Succinic acid can be synthesized into many vital compounds such as adipic acid, 2-pyrrolidone, 1, 4-butanediol, tetrahydrofuran, and degradable plastic PBS (Bi et al. 2018; Thakur et al. 2022). Traditionally, succinic acid has been chemically synthesized mainly from maleic acid or maleic anhydride (Dai et al. 2020), which relies on a petrochemical-based industrial production process system. However, the world’s heavy reliance on fossil fuels has led to greenhouse gas emissions and global warming (Zhong et al. 2023). The rising oil prices and environmental changes have led to the search for new green methods of succinic acid production (Thakur et al. 2022). The bio-based production of succinic acid could be performed by microbial fermentation with inexpensive raw materials (Dai et al. 2020), such as glycerol (Thanahiranya et al. 2023), hydrolysate of fruit and vegetable waste (Li et al. 2018a) and some green waste (Langsdorf et al. 2021). It is undoubtedly a more environmentally friendly way of production and can reduce greenhouse gas emissions. In recent years, many bacteria have been used for succinic acid production, including *Actinobacillus succinogenes* (Lee et al. 2022; Omwene et al. 2021), *Escherichia coli* (Valle et al. 2022; Li et al. 2018b) and *Corynebacterium glutamicu* (Fukui et al. 2019; Mao et al. 2018). Among them, natural succinic acid producers such as *A. succinogenes* can achieve higher yields (Choi et al. 2016). However, they require complex media composition, which undoubtedly increases the cost of the production of succinic acid (Wang et al. 2011). *E. coli* has the advantages of a fast growth rate, simple genetic manipulation, and low nutritional requirements. Therefore, it is considered one of the most promising metabolic engineering strains for the production of succinic acid in recent years (Li et al. 2017; Zhu et al. 2017).

Metabolic engineering modification is a common strategy used by researchers to improve the production of succinic acid. Overexpression of key node genes can effectively increase the yield of succinic acid (Li et al. 2016). Inactivation of the genes related to the byproduct pathway can greatly reduce the production of byproducts, then further promote the yield of succinic acid (Jantama et al. 2008). For example, the deletions of the *ldhA* and *pflB* genes are common means of promoting succinic acid production in *E. coli*, because of blocking the byproduct pathways such as formate as well as lactate (Wang et al. 2011; Yu et al. 2016; Zhu et al. 2016). Introduction of regulatory factors is also an effective method to increase the production of succinic acid (Zhang et al. 2018). In addition, increasing the supply of reducing power can improve the conversion of succinic acid. An engineered *E. coli* strain with an increasing supply of reducing power can yield succinic acid as high as 1.5 mol/mol (Zhu et al. 2014). Some major *E. coli* strains genetically modified for reducing byproducts, reducing PEP consumption, or global regulation were also studied for succinic acid production (Table S1).

*E. coli* MLB strains can hardly grow on glucose under anaerobic conditions for lacking genes encoding pyruvate formate lyase (PFL) and lactate dehydrogenase (LDH). While previous research proposes that its ability to metabolize glucose and produce succinic acid can be restored with acetate as a carbon source in the aerobic stage ( Li et al. 2017; Wu et al. 2007; Wu et al. 2012). The activity of enzymes related to succinate synthesis could be highly induced by acetate within the culture medium ( Wu et al. 2007). Incubation of cells in acetate medium during the aerobic phase is designed to induce the enzyme activity related to succinate synthesis, which would increase succinate production during the anaerobic phase. Furthermore, we hope that continuous acetate stimulation can promote the rapid growth of cells in acetate with increased activities of intracellular succinic acid-related metabolic enzymes, so as to achieve the purpose of increasing succinic acid production.

Adaptive laboratory evolution (ALE) is a technique for selecting strains with a better phenotype by long-term culture under a specific selection pressure or growth environment (Wang et al. 2022). With the development in recent decades, ALE can be used at the laboratory level to improve many abilities of microbes, such as promoting product yield (Kim et al. 2022; Godara and Kao 2021), increasing substrate utilization (Sun et al. 2023), improving growth rate (Barten et al. 2022), and enhancing tolerance (Wang et al. 2021). In addition to strain improvement, it has also been used to expand intracellular regulatory networks by revealing potential mechanisms that regulate cellular metabolism (SuRin and Pil 2020). The *E. coli* k-12 mutant strain, lacking the *ptsG* gene, had poor glucose utilization ability, but its ALE evolution strain could grow rapidly 48 h after the incubation on a glucose-containing medium (Kim et al. 2020). It can be said that ALE provides a reverse engineering approach that can overcome the shortcomings of metabolic engineering (Shepelin et al. 2018). The evolution of *E. coli* in an acetate environment has been reported for a long time. Noh et al. (2018) obtained a high tolerance *E. coli* strain to acetate by performing ALE in a medium containing 10 g/L acetate. Seong et al. (2020) harvested an evolved *E. coli* strain with high ATP production ability when using acetate as the sole carbon and energy source. Although some research has been reported on the *E. coli* ALE of acetate, there is currently no research on the application of acetate ALE to succinic acid production.

In this work, *E. coli* strain MLB, a strain derived from *E. coli* MG1655 with deletion of *ldhA* and *pflB*, was used as a starting strain of ALE. *E. coli* MG1655 has clear genetic background, require simple nutrition of the medium, and commonly be used in studies related to biochemical production. ALE was carried out in the medium containing different concentrations of acetate. After extensive screening, we ultimately obtained a succinic acid producer, strain MLB 46 − 05, which was more tolerant to high concentrations of substrates and product environments. The transcriptome comparison analysis was also performed between strain MLB and MLB 46 − 05 to reveal the possible mechanism of increasing succinate production.

## Methods and materials

### Strains and media

The starting strain MLB used in this study was derived from *E. coli* K12 strain MG1655 deleting *ldhA* and *pflB*. The strains used in this study are listed in Table S2. All of them were stored in 50% (w/w) glycerol at -80 °C. The adaptive laboratory evolution of the strains was carried out in M9 medium containing sodium acetate (pH 7). M9 medium: 15.12 g/L Na_2_HPO_4_.12H_2_O, 3 g/L KH_2_PO_4_, 0.5 g/L NaCl, 1 g/L NH_4_Cl, 0.011 g/L CaCl_2_, 0.1 mL/L trace element mixture, 0.2 mL/L 1%VB_1_, 0.5 g/L MgSO_4_.7H_2_O. For the fermentation experiments, Luria Bertani (LB) medium was used for strain activation, and the acetate-added medium (M9 with 10 g/L NH_4_Ac) was used for the aerobic phase of strain growth. The pH of acetate-added medium was 7.0. When the strain MLB grew in the aerobic stage, an additional 2 g/L YE would be added to the acetate-added medium. The anaerobic phase of succinic acid production was carried out using an anaerobic fermentation medium containing 3.78 g/L Na_2_HPO_4_.12H_2_O, 0.75 g/L KH_2_PO_4_, 0.5 g/L NaCl, 0.011 g/L CaCl_2_, 0.2 mL/L 1%VB_1_, 0.5 g/L MgSO_4_.7H_2_O, 0.1 mL/L trace element mixture, with additional addition of sterilized glucose and magnesium carbonate powder, the pH was adjusted to about 8.0.

### Adaptive laboratory evolution on acetate

Single colonies of the MLB strain were inoculated into 3 mL of LB medium at 37 °C overnight. Then 2% (v/v) of the culture was transferred into 250 mL shake flasks containing 50 mL of M9 medium with 10 g/L NaAc. The shake flasks were incubated at 37 °C and 220 rpm until the OD_600_ reached 3–4, and then 2% (v/v) of the culture was transferred into the same fresh medium. The passaging process was performed continuously until the improved growth rate became stable. Then, the NaAc concentration was increased to 20 g/L. The steps were repeated during the adaptive laboratory evolution (ALE). Single colonies of the evolved strain were inoculated into 3 mL of LB medium overnight at 37 °C and then placed in deep-well plates containing the evolved medium at an inoculum of 2% (v/v), and the growth status was detected by Bioscreen (Oy Growth Curves Ab Ltd, Finland).

## Two-stage fermentation in shake flask

Single colonies from fresh plates were inoculated into 3 mL LB medium and incubated at 37 °C, 220 rpm for 12 h. Primary pre-cultures were transferred to 250 mL shake flasks containing 50 mL of LB medium at an inoculum of 2% (V/V) and incubated at 37 °C, 220 rpm for 12 h. 2 mL of the secondary pre-culture was transferred to 100 mL of acetate-added medium (containing 10 g/L NH_4_Ac) for aerobic growth until the OD_600_ reached 4–5. Bacteria were collected by centrifuge and resuspended with the anaerobic fermentation medium. The concentrations of glucose in the anaerobic fermentation medium were different according to the different experiments. To investigate the effect of ALE on substrate inhibition during two-stage fermentation, 20, 30, 40, 60 and 80 g/L of initial concentrations of glucose were added separately in the different anaerobic fermentation. To investigate the effect of adaptive laboratory evolution on product inhibition during two-stage fermentation, different initial concentrations of sodium succinate (0, 20, 40, 60 and 80 g/L) were added in the anaerobic fermentation medium. The OD_600_ of the resuspended broth was concentrated to 10 or 20 according to the different experiments. The anaerobic condition for succinic acid production was achieved by blowing carbon dioxide flow for 1 min before sealing. The anaerobic flasks were incubated at 37 °C with 150 rpm shaking. There were three replicates of each experiment.

### High-density fermentation

The aerobic cultivation was the same as that in the section of “two-stage fermentation in shake flask”. In the anaerobic fermentation of high-density, the initial OD_600_ was increased to 40. About 80 g/L glucose was added initially and supplemented with about 60 g/L glucose 24 h after the fermentation, and the anaerobic flasks were incubated at 37 °C and shaken at 150 rpm.

### Analysis and detection methods

Cell growth was monitored at 600 nm (OD_600_) with a UV spectrophotometer (Xinmao, Shanghai, China) or a Bioscreen (Oy Growth Curves Ab Ltd, Finland). Succinic acid, glucose and mixed acids were determined by high-performance liquid chromatography (HPLC) equipped with a refractive index detector (RID) and a column (Bio-Rad Aminex® HPX-87 H, 300 × 7.8 mm, USA). The fermentation broth was centrifuged at 12,000 rpm for 10 min and then analyzed after filtrating through a 0.22 μm membrane. For the assay, the injection volume was 20 µL, the column temperature was 65℃, and the flow rate of 5 mM H_2_SO_4_ mobile phase was 0.6 mL/min.

### Transcriptome analysis

The strains were incubated aerobically in M9 medium containing 10 g/L NaAc for 24 h. Bacteria were collected by centrifugation for transcriptome sequencing. Transcriptome sequencing was completed by Guangzhou Gene Denovo Biotechnology Co. Raw read lengths were obtained by sequencing on the Illumina platform, and then PCA (Principal Component Analysis) was carried out using R. Differential analysis was performed using EdgeR and DESeq2 software.

## Results and discussion

### Effect of aerobic acetate metabolism on anaerobic succinic acid production

**Fig. 1:**
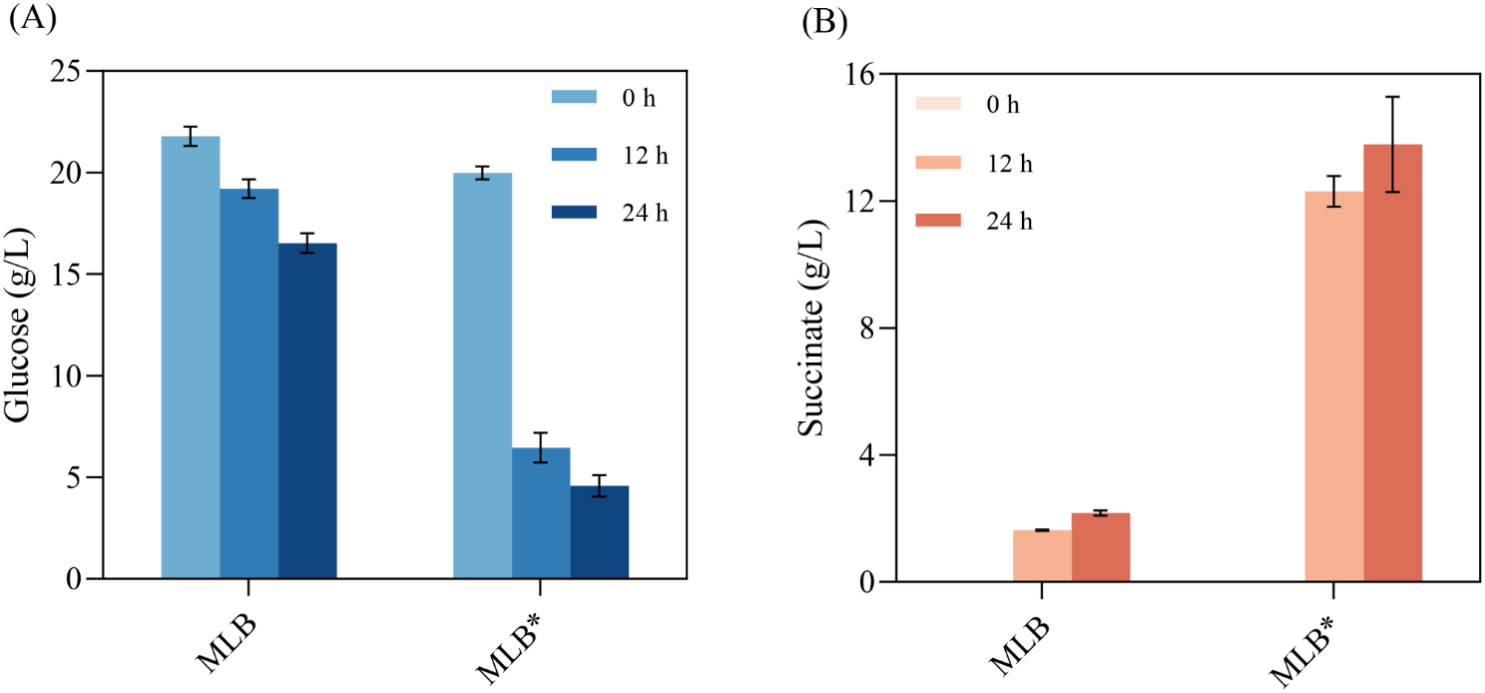
Effect of regulation of acetate metabolism on succinate production by two-stage fermentation of *E. coli* MLB. (**A**): glucose consumption in anaerobic fermentation; (**B**): succinate. MLB* refers to MLB grow on acetate

*E. coli* strain MLB is hard to metabolize glucose under anaerobic conditions due to the deletion of *ldhA* and *pflB* genes. In the two-stage fermentation, strain MLB also showed low glucose consumption and succinic acid production, when the carbon source was glucose in the aerobic stage. Similar as the previous study, the performance of glucose consumption and succinic acid production in anaerobic stage were enhanced significantly when the aerobic carbon source was changed to acetate (Fig. 1). In this experiment, the initial glucose added for anaerobic fermentation was 20 g/L. Only 5.25 g/L of glucose was consumed, and 2.17 g/L succinic acid was produced by strain glucose-grown MLB in 24 h (Fig. 1). Whereas, 15.40 g/L glucose was consumed, and 13.78 g/L succinate was produced by the acetate-grown MLB (Fig. 1). In the anaerobic stage, compared with those of the glucose-grown strain, the glucose consumption and succinate production rates of acetate-grown strain increased to 2.9 times and 6.3 times, respectively (Fig. 1). During the metabolic regulation of acetate utilization, the activity of enzymes related to succinate synthesis increased significantly, resulting in a greater flow of carbon flux to succinate synthesis (Oh et al. 2002; Wu et al. 2007). The related mechanisms were also explored in the subsequent section of transcriptome analysis. The results suggested that the metabolism regulation of acetate greatly facilitated the production of succinic acid in the two-stage fermentation of *E. coli* MLB. Therefore, the ALE on strain MLB was conducted in acetate utilization. We speculated that the evolved strains could undergo further changes on acetate metabolism that would be benefit for succinic acid production.

### Adaptive laboratory evolution of strain MLB

**Fig. 2:**
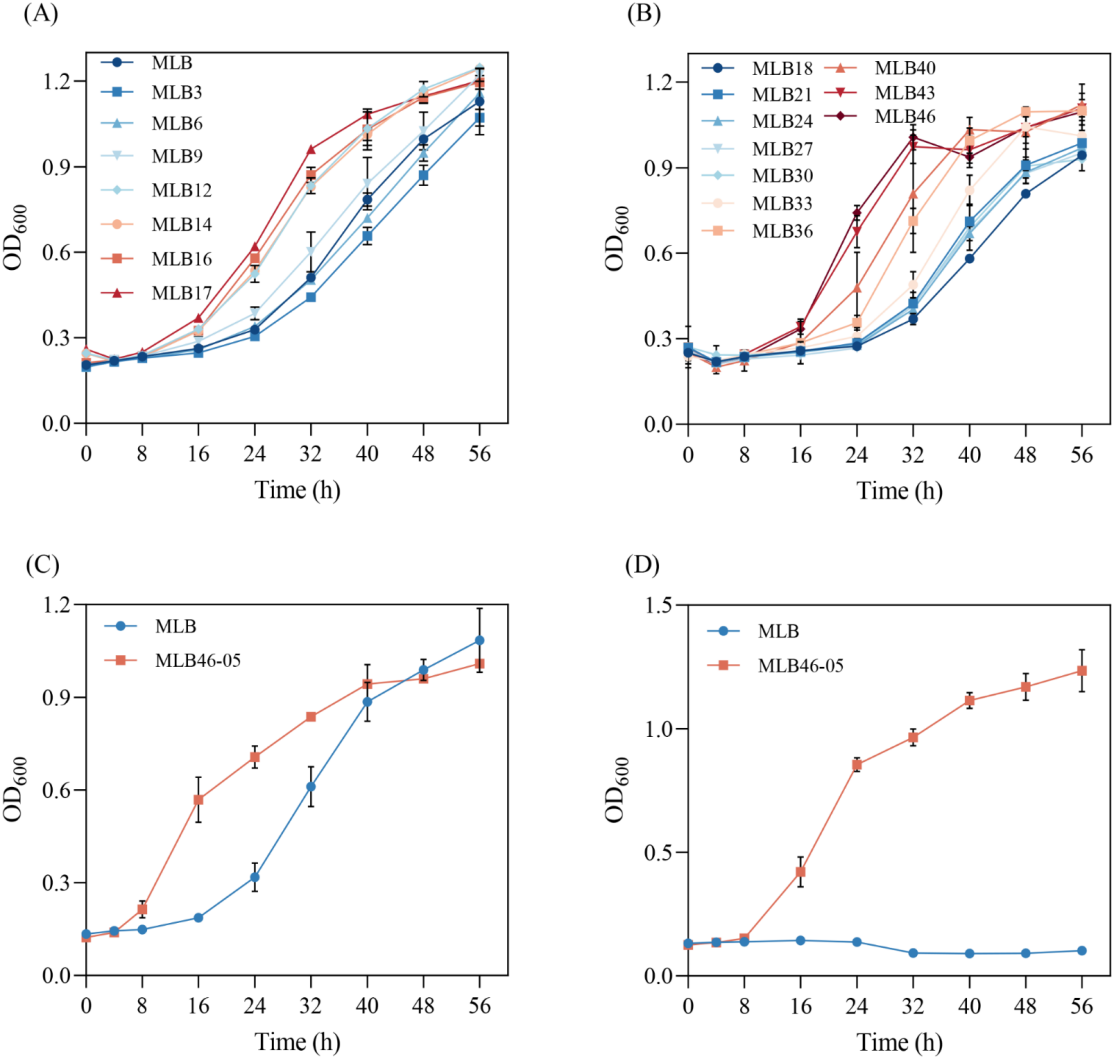
Adaptation laboratory evolution of *E. coli* in acetate with different concentrations. (**A**): 10 g/L sodium acetate; (**B**): 20 g/L sodium acetate; (**C**): growth of evolved *E. coli* in medium containing 10 g/L sodium acetate; (**D**): growth of evolved *E. coli* in medium containing 20 g/L sodium acetate

The ALE on strain MLB was performed in two levels. In the first level, strain MLB was evolved in a medium containing 10 g/L sodium acetate, and when the growth rate and the final biomass of the evolved strains tended to be stable, then enter the second level. In the second level, the concentration of sodium acetate was increased to 20 g/L. After two levels of evolution and screening, strain MLB46 was finally obtained, which could grow well in the M9 medium with 20 g/L sodium acetate (Fig. 2A and B). In the first level of ALE (10 g/L sodium acetate medium), OD_600_ of the starting strain MLB reached 1.13 after 56 h aerobic growth. The growth of MLB3 to MLB9 was slower and their specific growth rates were 0.049 h^− 1^, 0.049 h^− 1^, and 0.055 h^− 1^, respectively, whereas MLB12 showed an increase in maximum specific growth rate and was able to reach 0.058 h^− 1^ (Fig. 2A). Since the growth rate of MLB12 to MLB17 was significantly higher than that of MLB, the growth was stable and similar, and thereby ALE entered into the second level. The sodium acetate concentration was increased to 20 g/L, and the MLB18 (evolved from MLB17 in a 20 g/L sodium acetate) became the starting strain of the second level. The growth rate increased rapidly during the ALE experiments (Fig. 2B). When the growth rate finally stabilizes, the second level of ALE stopped at MLB46 (Fig. 2B). The evolved strain MLB46 had faster growth rates and higher biomass than the initial one.

Strain MLB46 was striped on a plate. Ten single colonies were chosen for the comparison of cell growth in the condition of 20 g/L sodium acetate. The single colony with the highest growth rate as well as biomass was obtained, and named as MLB46-05. The growth of strain MLB and MLB46-05 was further compared in acetate contained medium. The maximum specific growth rates of strains MLB and MLB46-05 were 0.082 h^− 1^ and 0.122 h^− 1^, respectively, in 10 g/L sodium acetate medium (Fig. 2C). In 20 g/L sodium acetate medium, the growth trends of the two strains were significantly different. The maximum specific growth rate of strain MLB46-05 could reach 0.127 h^− 1^, while strain MLB could hardly grow in this medium (Fig. 2D). It is probably because the higher concentration of acetate has a toxic effect on the unevolved *E. coli* (Pinhal et al. 2019). It was obvious that ALE could effectively improve the growth rate of *E. coli* using acetate as carbon source and their tolerance to acetate. The evolved strain MLB46-05 could adapt well to the medium with 20 g/L sodium acetate (Fig. 2D), which was even higher than the concentration of tolerant evolution reported by Noh et al. (2018), Seong et al. (2020) and so on.

### Transcriptome analysis of evolved strain

**Fig. 3:**
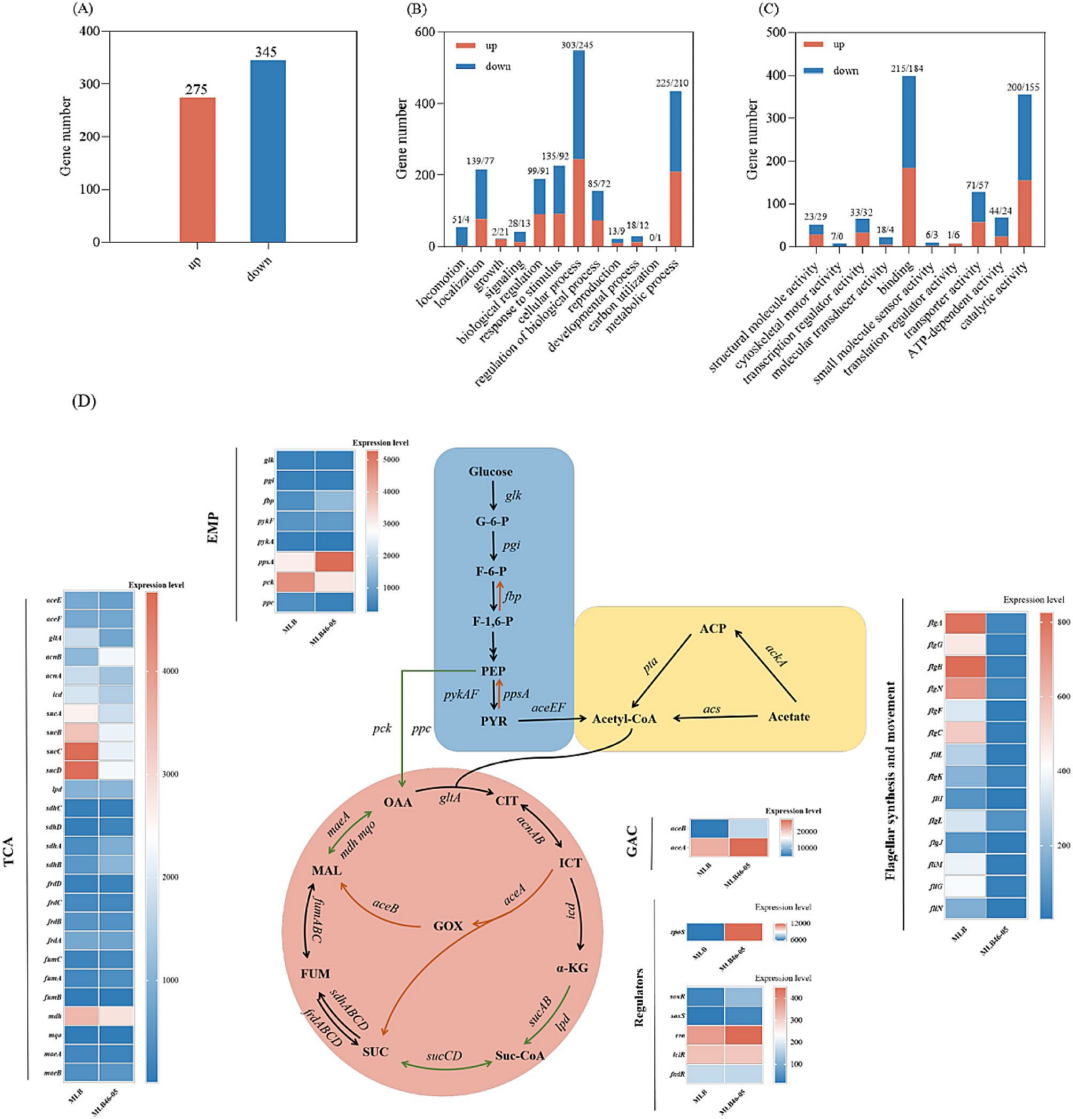
Difference in gene expression between MLB46-05 and MLB. (**A**): total amount of up- and down-regulated gene expression; (**B**): Biological Process gene expression difference; (**C**): Molecular Function gene expression difference. Up (red) indicates genes with up-regulated expression, down (blue) indicates genes with down-regulated expression, and the numbers x/y indicate down/up; (**D**): Effects of ALE on metabolic pathways and genes in *E. coli*. Abbreviations in the figure as well as gene annotations are available in the Table S3. Red arrows indicate that the expression of related genes in this pathway is significantly up-regulated (At least 1.2 times) and green arrows indicate that the expression of related genes in this pathway is down-regulated (At least 1.2 times)

After evolutionary passage, the phenotype of strain MLB46-05 was significantly different from strain MLB (Fig. 3A - C). Compared with strain MLB, 275 genes were up-regulated and 345 genes were down-regulated in strain MLB46-05. The number of down-regulated genes was higher than that of up-regulated genes. Previously, some research have mentioned that in relatively poor carbon sources, *E. coli* reduces the expression of biosynthetic genes to match lower growth rates and save energy (Oh et al. 2002). Similar conclusions can be drawn from the results of our experiments, where the analysis of genes related to biological processes suggests that cells in acetate minimize their vital activities to maintain basic growth.

Compared with the starting strain MLB, the *fbp*, and *ppsA* genes in the EMP pathway of the evolved strain MLB46-05 were up-regulated, while the expression levels of the *pck* and *ppc* genes were down-regulated (Fig. 3D). This indicated that the generated PEP flowed less towards OAA, possibly due to using acetate as the sole carbon source in the evolution, which has induced the gluconeogenesis pathway (Oh et al. 2002). Less carbon flow through OAA to TCA and the respiratory chain would result in less ROS in aerobic stage, which could alleviate the stress from acetate or osmotic pressure and let the cell grow better. Genes associated with acetate assimilation (e.g., *acs*, *pta*, and *ackA*) were reduced in MLB46-05 compared to MLB, and the mechanism of this phenomenon needs to be further studied (Fig. 3D). Compared with strain MLB, the expression levels of genes *aceA* and *aceB* in strain MLB46-05 were up-regulated to 1.2 and 2.7 times, respectively, indicating an enhanced glyoxylate cycle in the evolved strain possibly. Similar results were also observed by Seong et al. (2020), the expression of *aceA* and *aceB* genes was up-regulated in the strain undergoing ALE in acetate stress. Interestingly, in the tricarboxylic acid cycle (TCA), genes *mdh*, *sucA*, *sucB*, *sucC*, and *sucD* were down-regulated to varying degrees (Fig. 3D).

In addition, in strain MLB46-05, genes related to flagellar synthesis and motility were significantly down-regulated. The down-regulation gene expression levels related to flagellar synthesis and motility was the most significant among numerous gene expression changes. We speculated that it might be to adapt to the growth in acetate, and the flagella function of strain MLB46-05 deteriorates, reducing both motility and protein synthesis, which is beneficial for saving energy for cell growth. The expression level of regulatory factors, *rpoS*, *soxR*, and *soxS*, in strain MLB46-05 were upregulated by 2.1, 3.8, and 2.5 times (Fig. 3D). *rpoS* is a regulator for general stress response (a global regulator that regulates the expression of a variety of genes). *soxRS* is a regulator for the superoxide response, which contributes to superoxide scavenging in the cell. During the evolution with high concentrations of acetate, the gene expression levels of these regulators was upregulated to help cells against with the stress caused by acetate. The genes directly or indirectly regulated by *rpoS* factors include *fumC* in the TCA cycle, *talA* and *tktB* in the PP (pentose phosphate) pathway, all of which exhibited an up-regulation. *aceA* and *aceB* are positively regulated by global regulatory genes, such as *cra*, and negatively regulated by *iclR* and *fadR* (Rahman and Shimizu 2008; Kim et al. 2018). *cra* is up-regulated by 1.2-fold in terms of relative expression. There was essentially no difference in the expression of *iclR* and *fadR*. The gene expression of *aceAB* was also up-regulated under the regulation of *cra*. In addition, *ppsA* is also one of the genes regulated by *cra* gene, and *ppsA* gene expression also showed up-regulation. The results of transcriptome analysis showed that during evolution in a culture medium with acetate as the sole carbon source, the expression levels of genes related to flagella synthesis and motility were down-regulated, while the expression levels of genes for transcription factors resistant to abiotic stresses and antioxidant stress were up-regulated. This suggested that during the evolution of acetate, not only promoted its ability to reduce the toxic effects of superoxide, but also continuously enhanced its ability to adapt to abiotic stress (including acetate stress and osmotic stress caused by substrates and products), then could grow well in a stressful environment. At the same time, *E. coli* reduced its non-essential life activities, then the saving energy and carbon flow were redirected to the enhanced glyoxylate cycle and rTCA.

### Effect of aerobic ALE on anaerobic substrate inhibition in two-stage fermentation

A high concentration of substrate is a critical inhibition factor for microbial fermentation. To verify whether ALE in acetate has a positive effect on the production of succinic acid by *E. coli* in two-stage fermentation, strains MLB and MLB46-05 were inoculated in the medium containing 20, 40, 60, and 80 g/L glucose for two-stage fermentation. During the tests, as the glucose concentration in the medium was increased, the initial bacterial concentration, the amount of MgCO_3_ added during the anaerobic stage, and the length of the anaerobic fermentation stage were increased accordingly.

**Fig. 4:**
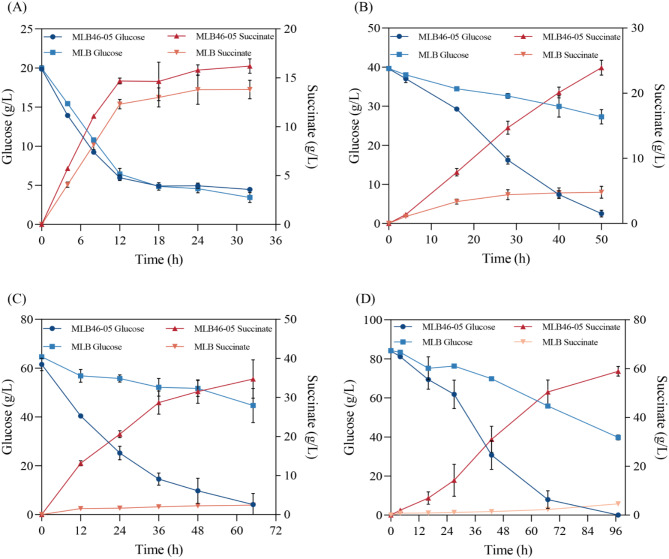
Effect of adaptive laboratory evolution on substrate inhibition during two-stage fermentation. (**A**): anaerobic fermentation in 20 g/L glucose with a cell concentration of 10 OD_600_; (**B**): anaerobic fermentation in 40 g/L glucose with a cell concentration of 10 OD_600_; (**C**): anaerobic fermentation in 60 g/L glucose with a cell concentration of 20 OD_600_; (**D**): anaerobic fermentation in 80 g/L glucose with a cell concentration of 20 OD_600_

All of the cells were grown in 10 g/L NH_4_Ac in the aerobic phase. When the glucose concentration was 20 g/L in the anaerobic condition, the initial bacterial concentration (OD_600_) of strains MLB and MLB46-05 during the anaerobic fermentation stage was enriched to 10. Within the first 12 h of the two strains, glucose was consumed rapidly, succinic acid was produced at a fast rate. The yield of succinic acid in MLB46-05 (15.89 g/L) was 17.7% higher than that of MLB (13.49 g/L) (Fig. 4A). In 30 h fermentation, the strains did not consume all glucose, which is speculated to be due to the low amount of MgCO_3_ added. MgCO_3_ is a well-performing pH regulator (Putri et al. 2020) that can neutralize the acid produced in anaerobic fermentation. Furthermore, MgCO_3_ is not only able to form the CO_2_ required for succinic acid production but Mg^2+^ is also a cofactor for the enzyme (Bazaes et al. 2007).

In fermentation with 40, 60, and 80 g/L glucose as substrate, the succinic acid yields of the evolved strain MLB46-05 were 23.92 g/L, 34.70 g/L, and 58.89 g/L, respectively, which were 5-fold, 14.4-fold, and 12.9-fold higher than those of strain MLB (Fig. 4B-D). A kinetic model for the production of succinic acid with *E. coli* strain KJ12201 from xylose and glucose is proposed based on Monod’s equation. It reveals that xylose has a strong inhibitory effect on the growth rate of strain KJ12201, while glucose does not (Chaleewong et al. 2022). The results of this study showed that high concentrations of glucose had a strong inhibitory effect on unevolved *E. coli* strain MLB. Overall, the ALE in acetate showed a positive effect on *E. coli* to the substrate inhibition in two-stage fermentation and further promoted its succinate production.

### Effect of aerobic ALE on anaerobic succinate inhibition during two-stage fermentation

From the aforementioned experiments, we found that the evolved strain MLB46-05 was not only had a higher tolerance to high concentrations of substrates but also had better production efficiency. However, in actual fermentation production, the strain not only has to withstand the pressure from substrate but also needs to tolerate the pressure caused by product accumulation. To verify whether the lab-evolved strains were more resistant to product inhibition, additional 0, 20, 40, 60, and 80 g/L sodium succinate was added in the anaerobic phase of two-stage fermentation, respectively. All of the initial OD_600_ of MLB and MLB46-05 were concentrated to 10 in the anaerobic fermentations.

**Fig. 5:**
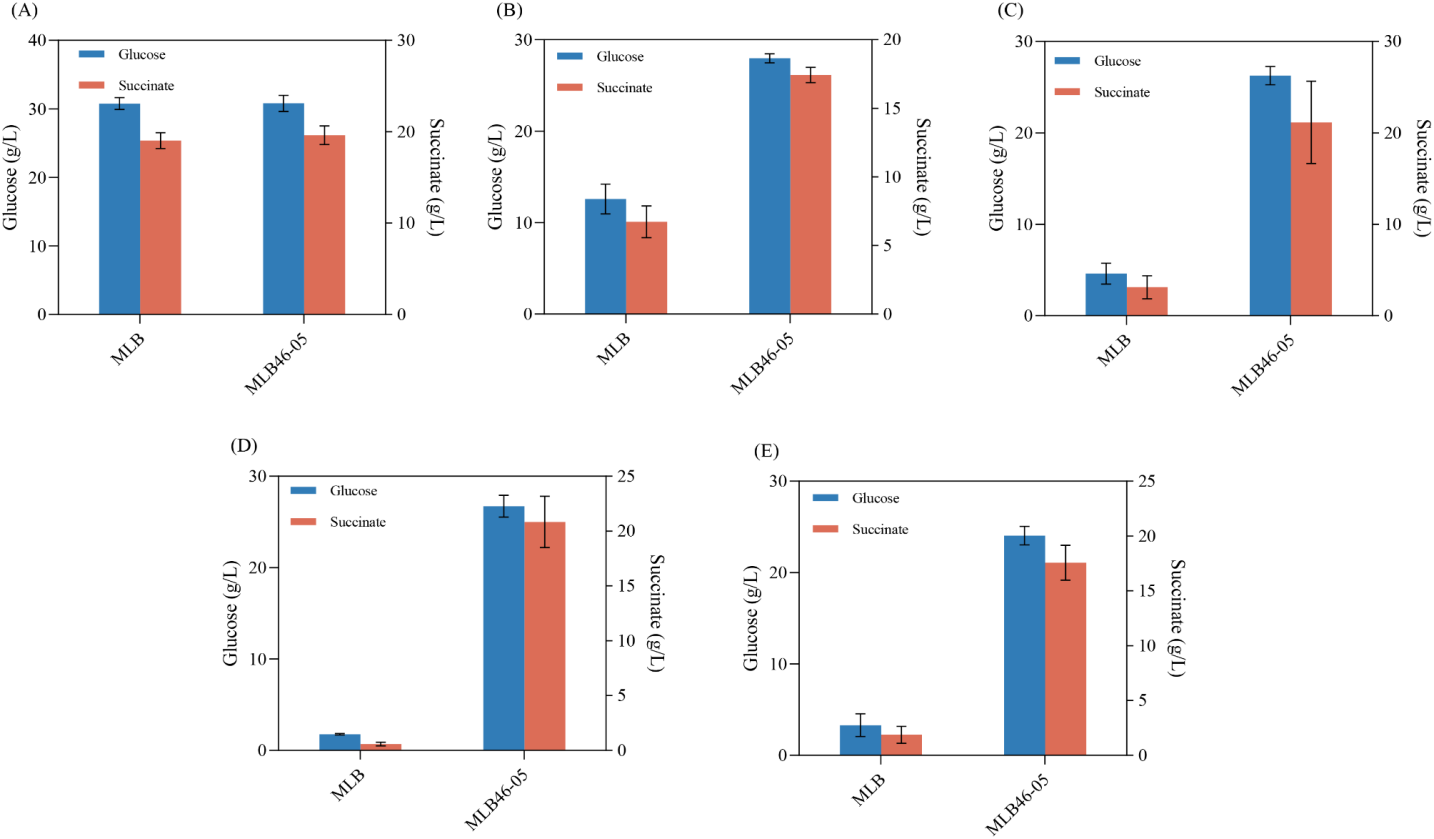
Effect of adaptive laboratory evolution on product inhibition during two-stage fermentation. (**A**): glucose consumption and succinic acid production in anaerobic fermentation with addition of 0 g/L sodium succinate; (**B**): glucose consumption and succinic acid production in anaerobic fermentation with addition of 20 g/L sodium succinate; (**C**): glucose consumption and succinic acid production in anaerobic fermentation with addition of 40 g/L sodium succinate; (**D**): glucose consumption and succinic acid production in anaerobic fermentation with addition of 60 g/L sodium succinate; (**E**): glucose consumption and succinic acid production in anaerobic fermentation with addition of 80 g/L sodium succinate

When anaerobic fermentation was conducted in a control medium just containing 30 g/L glucose without additional succinate for 48 h, it resulted in similar succinic acid production by strains MLB46-05 and MLB (19.61 and 19.03 g/L respectively) (Fig. 5A). When additional succinate was added, the significant differences in succinic acid production and substrate consumption between the two strains could be detected.

When additional amounts of sodium succinate were added at concentrations of 20, 40, 60, and 80 g/L (as shown in Fig. 5B-E), the production of succinic acid by the strain MLB46-05 was approximately 17.0–21.0 g/L. Compared with the control group (without additional succinate), the decrease in succinate production by strain MLB46-05 was less than 12.0%, while the decrease in succinate production by strain MLB was about 64.0–97.0% lower compared to the control group. Lin et al. (2008) found that the growth of *A. succinogenes* ATCC 55,618 was completely inhibited at a succinate content of 45.6 g/L, and a similar phenomenon was also observed in *E. coli* (Andersson et al. 2009). In this study, MLB almost lost the ability to metabolize glucose for succinic acid production in environment of high initial succinate concentration, probably due to the hyperosmotic stress brought by the high concentration of sodium succinate which deprived MLB of its normal metabolic capacity. On the contrary, strain MLB46-05 evolved in sodium acetate not only adapted to acetate but also developed tolerance to high concentrations of succinate. Tolerance to acid may be related to resistance mechanisms, and increased expression of genes for resistance can help cells grow well under hyper acidic conditions.

### Results of high-density fermentation studies

**Fig. 6:**
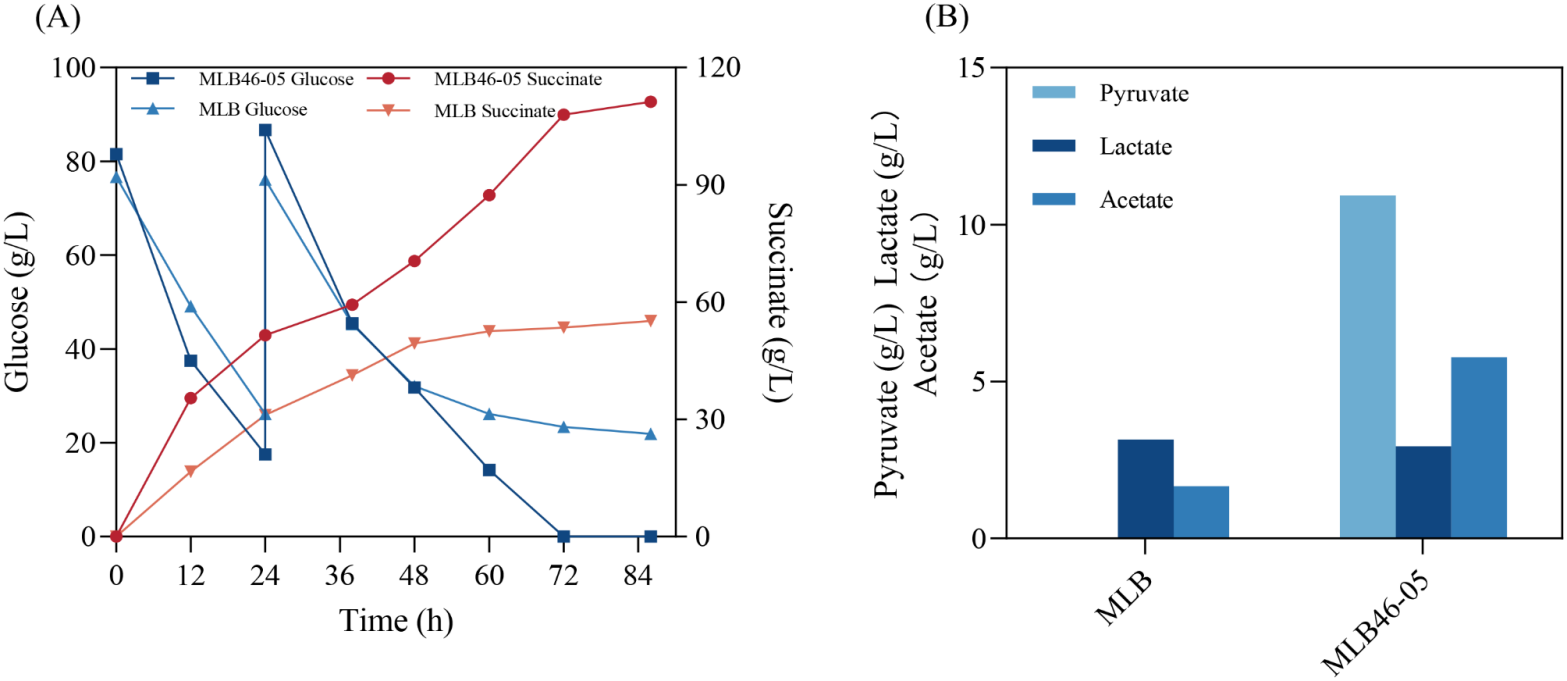
High-density anaerobic transformation of strains. (**A**): high-density fermentation process of strains MLB and MLB46-05; (**B**): accumulation of by-products

High-density fermentation comparisons between pre and post-evolution strains were conducted. During the 86 h fermentation process in shake flask, strain MLB could consume approximately 104.68 g/L of glucose and produce 55.16 g/L of succinic acid, while strain MLB46-05 could deplete all glucose and produce 111 g/L of succinic acid (Fig. 6A). Compared to other studies, the production obtained in this study is relatively high (Table S1). A certain amount of by-products was also found to be accumulated during the fermentation process (Fig. 6B). Strain MLB46-05 accumulated 10.93 g/L pyruvic acid, 2.94 g/L lactic acid, and 5.78 g/L acetate, whereas strain MLB accumulated 3.15 g/L lactic acid, as well as 1.67 g/L acetate, and no pyruvic acid production was detected. The succinic acid conversion rate of strain MLB46-05 reached 0.74 g/g glucose, which was slightly lower than the results of Guo et al. (2022) and Yu et al. (2016). But according to previous studies, conversion rates could be further improved by optimization of fermentation conditions or through metabolic engineering methods (Li et al. 2016; Crigler et al. 2018). Because strain MLB46-05 possessed stronger resistance to the substrate and product inhibition than the original strain MLB by ALE. It could grow well and produce more succinic acid than strain MLB in high-density fermentations. The succinic acid titer of strain MLB46-05 was 101.7% higher than that of MLB. Based on the transcriptome, we analyzed that the down-regulation of *sucABCD* gene expression and the up-regulation of *aceAB* gene expression could be the reason why strain MLB46-05 was able to obtain higher succinic acid yields than strain MLB when entering anaerobic fermentation in a two-stage fermentation. Moreover, the increased tolerance of strain MLB46-05 to acetic acid also allowed it to tolerate higher concentrations of succinic acid as well as glucose, which may also be one of the reasons for its higher yield than that of MLB.

## Conclusions

In this study, the evolved strain MLB46-05 was obtained by adaptive evolution on acetate. It has a high tolerance on acetate in the aerobic stage as well as a high succinic acid production rate in the anaerobic stage. In high-density fermentation, it produced 111 g/L succinic acid about two times of the control one. Transcriptome comparison analysis revealed that the expression of genes related to the gluconeogenesis pathway, the glyoxylate cycle and some stress-tolerance factors were up-regulated in the evolved strain. Overall, aerobically ALE could be used to fill the gap of metabolic engineering in anaerobic biosynthesis.

### Electronic supplementary material

Below is the link to the electronic supplementary material.

Supplementary Material 1

Supplementary Material 2

## Data Availability

Data maybe made available on request.

## References

[CR1] Andersson C, Helmerius J, Hodge D, Berglund KA, Rova U (2009). Inhibition of succinic acid production in metabolically engineered *Escherichia coli* by neutralizing agent, organic acids, and osmolarity. Biotechnol Prog.

[CR2] Barten R, van Workum D-JM, de Bakker E, Risse J, Kleisman M, Navalho S, Smit S, Wijffels RH, Nijveen H, Barbosa MJ (2022). Genetic mechanisms underlying increased microalgal thermotolerance, maximal growth rate, and yield on light following adaptive laboratory evolution. BMC Biol.

[CR3] Bazaes S, Toncio M, Laivenieks M, Zeikus JG, Cardemil E (2007). Comparative kinetic effects of Mn (II), mg (II) and the ATP/ADP ratio on Phosphoenolpyruvate Carboxykinases from *Anaerobiospirillum succiniciproducens* and *Saccharomyces cerevisiae*. Protein J.

[CR4] Bi S, Tan B, Soule JL, Sobkowicz MJ (2018). Enzymatic degradation of poly (butylene succinate-co-hexamethylene succinate). Polym Degrad Stab.

[CR5] Chaleewong T, Khunnonkwao P, Puchongkawarin C, Jantama K (2022). Kinetic modeling of succinate production from glucose and xylose by metabolically engineered *Escherichia coli* KJ12201. Biochem Eng J.

[CR6] Choi S, Song CW, Shin JH, Lee SY (2015). Biorefineries for the production of top building block chemicals and their derivatives. Metab Eng.

[CR7] Choi S, Song H, Lim SW, Kim TY, Ahn JH, Lee JW, Lee M-H, Lee SY (2016). Highly selective production of succinic acid by metabolically engineered *Mannheimia succiniciproducens* and its efficient purification. Biotechnol Bioeng.

[CR8] Crigler J, Bannerman-Akwei L, Cole AE, Eiteman MA, Altman E (2018). Glucose can be transported and utilized in *Escherichia coli* by an altered or overproduced N-acetylglucosamine phosphotransferase system (PTS). Microbiology.

[CR9] Dai Z, Guo F, Zhang S, Zhang W, Yang Q, Dong W, Jiang M, Ma J, Xin F (2020). Bio-based succinic acid: an overview of strain development, substrate utilization, and downstream purification. Biofuels Bioprod Biorefin.

[CR10] Fukui K, Nanatani K, Nakayama M, Hara Y, Tokura M, Abe K (2019). *Corynebacterium glutamicum* CgynfM encodes a dicarboxylate transporter applicable to succinate production. J Biosci Bioeng.

[CR11] Godara A, Kao KC (2021). Adaptive laboratory evolution of β-caryophyllene producing *Saccharomyces cerevisiae*. Microb Cell Fact.

[CR12] Guo F, Wu M, Zhang S, Feng Y, Jiang W, Xin F, Zhang W, Jiang M (2022). Improved succinic acid production through the reconstruction of methanol dissimilation in *Escherichia coli*. Bioresour Bioprocess.

[CR13] Jantama K, Zhang X, Moore JC, Shanmugam KT, Svoronos SA, Ingram LO (2008). Eliminating side products and increasing succinate yields in engineered strains of *Escherichia coli* C. Biotechnol Bioeng.

[CR14] Kim D, Seo SW, Gao Y, Nam H, Guzman GI, Cho B-K, Palsson BO (2018). Systems assessment of transcriptional regulation on central carbon metabolism by Cra and CRP. Nucleic Acids Res.

[CR15] Kim HJ, Jeong H, Lee SJ (2020). Short-term adaptation modulates anaerobic metabolic flux to succinate by activating ExuT, a novel D-glucose transporter in *Escherichia coli*. Front Microbiol.

[CR16] Kim K, Hou CY, Choe D, Kang M, Cho S, Sung BH, Lee D-H, Lee S-G, Kang TJ, Cho B-K (2022). Adaptive laboratory evolution of *Escherichia coli* W enhances gamma-aminobutyric acid production using glycerol as the carbon source. Metab Eng.

[CR17] Langsdorf A, Volkmar M, Holtmann D, Ulber R (2021). Material utilization of green waste: a review on potential valorization methods. Bioresour Bioprocess.

[CR18] Lee J-S, Lin C-J, Lee W-C, Teng H-Y, Chuang M-H (2022). Production of succinic acid through the fermentation of *Actinobacillus succinogenes* on the hydrolysate of Napier grass. Biotechnol Biofuels Bioprod.

[CR20] Li Q, Wu H, Li Z, Ye Q (2016). Enhanced succinate production from glycerol by engineered *Escherichia coli* strains. Bioresour Technol.

[CR21] Li Q, Huang B, Wu H, Li Z, Ye Q (2017). Efficient anaerobic production of succinate from glycerol in engineered *Escherichia coli* by using dual carbon sources and limiting oxygen supply in preceding aerobic culture. Bioresour Technol.

[CR19] Li C, Yang X, Gao S, Chuh AH, Lin CSK (2018a) Hydrolysis of fruit and vegetable waste for efficient succinic acid production with engineered *Yarrowia Lipolytica*. J Clean Prod 179:151–159. 10.1016/j.jclepro.2018.01.081

[CR22] Li Q, Huang B, He Q, Lu J, Li X, Li Z, Wu H, Ye Q (2018). Production of succinate from simply purified crude glycerol by engineered *Escherichia coli* using two-stage fermentation. Bioresour Bioprocess.

[CR23] Lin SKC, Du C, Koutinas A, Wang R, Webb C (2008). Substrate and product inhibition kinetics in succinic acid production by *Actinobacillus succinogenes*. Biochem Eng J.

[CR24] Mao Y, Li G, Chang Z, Tao R, Cui Z, Wang Z, Tang Y-j, Chen T, Zhao X (2018). Metabolic engineering of *Corynebacterium glutamicum* for efficient production of succinate from lignocellulosic hydrolysate. Biotechnol Biofuels.

[CR25] Noh MH, Lim HG, Woo SH, Song J, Jung GY (2018). Production of itaconic acid from acetate by engineering acid-tolerant *Escherichia coli* W. Biotechnol Bioeng.

[CR26] Oh M-K, Rohlin L, Kao KC, Liao JC (2002). Global expression profiling of acetate-grown *Escherichia coli*. J Biol Chem.

[CR27] Omwene PI, Yağcıoğlu M, Öcal-Sarihan ZB, Ertan F, Keris-Sen ÜD, Karagunduz A, Keskinler B (2021). Batch fermentation of succinic acid from cheese whey by *Actinobacillus succinogenes* under variant medium composition. 3 Biotech.

[CR28] Pinhal S, Ropers D, Geiselmann J, Jong H (2019). Acetate metabolism and the inhibition of bacterial growth by acetate. J Bacteriol.

[CR29] Putri DN, Sahlan M, Montastruc L, Meyer M, Negny S, Hermansyah H (2020). Progress of fermentation methods for bio-succinic acid production using agro-industrial waste by *Actinobacillus succinogenes*. Energy Rep.

[CR30] Rahman M, Shimizu K (2008). Altered acetate metabolism and biomass production in several *Escherichia coli* mutants lacking rpos-dependent metabolic pathway genes. Mol Biosyst.

[CR31] Seong W, Han GH, Lim HS, Baek JI, Kim S-J, Kim D, Kim SK, Lee H, Kim H, Lee S-G, Lee D-H (2020). Adaptive laboratory evolution of *Escherichia coli* lacking cellular byproduct formation for enhanced acetate utilization through compensatory ATP consumption. Metab Eng.

[CR32] Shepelin D, Hansen AS, Lennen R, Luo H, Herrgård MJ (2018). Selecting the best: evolutionary engineering of chemical production in microbes. Genes.

[CR33] Sun Q, Liu D, Chen Z (2023). Engineering and adaptive laboratory evolution of *Escherichia coli* for improving methanol utilization based on a hybrid methanol assimilation pathway. Front Bioeng Biotechnol.

[CR34] SuRin L, Pil K (2020). Current status and applications of adaptive laboratory evolution in industrial microorganisms. J Microbiol Biotechnol.

[CR35] Thakur S, Chaudhary J, Singh P, Alsanie WF, Grammatikos SA, Thakur VK (2022). Synthesis of bio-based monomers and polymers using microbes for a sustrainable bioeconomy. Bioresour Technol.

[CR36] Thanahiranya P, Charoensuppanimit P, Sadhukhan J, Soottitantawat A, Arpornwichanop A, Thongchul N, Assabumrungrat S (2023). Succinic acid production from glycerol by *Actinobacillus succinogenes*: Techno-economic, environmental, and exergy analyses. J Clean Prod.

[CR37] Valle A, Soto Z, Muhamadali H, Hollywood KA, Xu Y, Lloyd JR, Goodacre R, Cantero D, Cabrera G, Bolivar J (2022). Metabolomics for the design of new metabolic engineering strategies for improving aerobic succinic acid production in *Escherichia coli*. Metabolomics.

[CR38] Wang D, Li Q, Song Z, Zhou W, Su Z, Xing J (2011). High cell density fermentation via a metabolically engineered *Escherichia coli* for the enhanced production of succinic acid. J Chem Technol Biotechnol.

[CR40] Wang Z, Zhou L, Lu M, Zhang Y, Perveen S, Zhou H, Wen Z, Xu Z, Jin M (2021). Adaptive laboratory evolution of *Yarrowia Lipolytica* improves ferulic acid tolerance. Appl Microbiol Biotechnol.

[CR39] Wang G, Li Q, Zhang Z, Yin X, Wang B, Yang X (2022). Recent progress in adaptive laboratory evolution of industrial microorganisms. J Ind Microbiol Biotechnol.

[CR41] Wu H, Li Z, Zhou L, Ye Q (2007). Improved succinic acid production in the anaerobic culture of an *Escherichia coli pflB ldhA* double mutant as a result of enhanced anaplerotic activities in the preceding aerobic culture. Appl Environ Microbiol.

[CR42] Wu H, Li Q, Li Z, Ye Q (2012). Succinic acid production and CO_2_ fixation using a metabolically engineered *Escherichia coli* in a bioreactor equipped with a self-inducing agitator. Bioresour Technol.

[CR43] Yu J-H, Zhu L-W, Xia S-T, Li H-M, Tang Y-L, Liang X-H, Chen T, Tang Y-J (2016). Combinatorial optimization of CO_2_ transport and fixation to improve succinate production by promoter engineering. Biotechnol Bioeng.

[CR44] Zhang W, Zhu J, Zhu X, Song M, Zhang T, Xin F, Dong W, Ma J, Jiang M (2018). Expression of global regulator IrrE for improved succinate production under high salt stress by *Escherichia coli*. Bioresour Technol.

[CR45] Zhong W, Li H, Wang Y (2023). Design and construction of artificial biological systems for one-carbon utilization. Biodes Res.

[CR46] Zhu L-W, Tang Y-J (2017). Current advances of succinate biosynthesis in metabolically engineered *Escherichia coli*. Biotechnol Adv.

[CR47] Zhu X, Tan Z, Xu H, Chen J, Tang J, Zhang X (2014). Metabolic evolution of two reducing equivalent-conserving pathways for high-yield succinate production in *Escherichia coli*. Metab Eng.

[CR48] Zhu L-W, Xia S-T, Wei L-N, Li H-M, Yuan Z-P, Tang Y-J (2016). Enhancing succinic acid biosynthesis in *Escherichia coli* by engineering its global transcription factor, catabolite repressor/activator (cra). Sci Rep.

