# Supplementary material for: Effect of adaptive laboratory evolution of engineered Escherichia coli in acetate on the biosynthesis of succinic acid from glucose in two-stage cultivation
Source: Bioresour Bioprocess. 2024 Apr 5;11(1):34. doi: 10.1186/s40643-024-00749-5 (PMC10997558; doi:10.1186/s40643-024-00749-5)
Supplement: Supplementary file 1 — Supplementary Material 1 [file 40643_2024_749_MOESM1_ESM.docx]

***Supporting Information***

**Effect of adaptive laboratory evolution of engineered *Escherichia coli* in acetate on the biosynthesis of succinic acid from glucose in two-stage cultivation**

Jiaping Jiang^1#^, Yuanchan Luo^1#^, Peng Fei^1^, Zhengtong Zhu^1^,Jing Peng^1^, Juefeng Lu^1^, Du Zhu^4^, Hui Wu*^,1,2,3^

^1^ State Key Laboratory of Bioreactor Engineering, Shanghai Frontiers Science Center of Optogenetic Techniques for Cell Metabolism, School of Biotechnology, East China University of Science and Technology, 130 Meilong Road, Shanghai 200237, China

^2^ MOE Key Laboratory of Bio-Intelligent Manufacturing, School of Bioengineering, Dalian University of Technology, Dalian, China.

^3^ Shanghai Collaborative Innovation Center for Biomanufacturing Technology, 130 Meilong Road, Shanghai 200237, China

^4^ Key Lab of Bioprocess Engineering of Jiangxi Province, College of Life Sciences, Jiangxi Science and Technology Normal University, Nanchang, 330013, China

* Corresponding author: Hui Wu

Telephone: +86-21-64253701

Fax: +86-21-64252250

E-mail: [hwu@ecust.edu.cn](mailto:hwu@ecust.edu.cn)

**Table S1 Current status of studies on succinic acid production by *E. coli* fermentation**

| **Strains** | **Genotype** | **Substrate** | **Fermentation** | **Titer**  **(g/L)** | **Yield**  **(g/g)** | **References** |
| --- | --- | --- | --- | --- | --- | --- |
| *E. coli* W1485 | *ΔpflB::Cm*, *ΔldhA::Kan*, *ΔptsG*, expression of *E. coli cra* gene | Glucose | Dual-phase fed-batch | 79.8 | 0.78 | (Zhu et al., 2016) |
| *E. coli* SD121 | Expression of *ppc*, deletion of *pflB*, *ldhA* and *ptsG* | Glucose | Dual-phase fed-batch | 116.2 | 1.13 | (Wang et al., 2011) |
| *E. coli* AFP111 | Deletion of *pflB*, *ldhA* and *ptsG* | Glucose | Dual-phase fed-batch | 101.2 | 1.07 | (Jiang et al., 2010) |
| *E. coli* BE062 | Expression of global regulator IrrE | Glucose | Anaerobic fed-batch | 24.5 | 0.88 | (Zhang et al., 2018) |
| *E. coli* JW1021 | A novel riboregulator switch | Glucose | Dual-phase fed-batch | 114.0 | 0.91 | (Wang et al., 2018) |
| *E. coli* NZN111 | Deletion of *ldhA* and *pflB* | Acetate glucose | Dual-phase fed-batch | 60.1 | - | (Liu et al., 2011) |
| *E. coli* Tang1527 | Expression of *bicA*, *sbtA*, *ppc* and *pck*; deletion of *pflB*, *ldhA* and *ptsG* | Glucose | Dual-phase fed-batch | 89.4 | 0.83 | (Yu et al., 2016) |
| *E. coli* YY-GS004 | Δ*ptsI*, Δ*ldhA*, Δ*pflB*, Ppck*-*galp*, Ppck*-*pck*, Δ*glpK*, Δ*dhaKLM*::M1-93-RBSL-4-*dhaK* | Glycerol | Anaerobic fed-batch | 57.0 | 1.18 | (Yu et al., 2019) |

**Table S2 Strains used in this study**

| **Strains** | **Description** | **Source** |
| --- | --- | --- |
| MLB | *E. coli* K-12 substr.MG1655 Δ*ldhA::FRT* Δ*pflB::FRT* | Laboratory collection |
| MLB3 | From the culture of 3 times of transition in the adaptive laboratory evolution in 10 g/L NaAc from MLB | This study |
| MLB6 | From the culture of 6 times of transition in the adaptive laboratory evolution in 10 g/L NaAc from MLB | This study |
| MLB9 | From the culture of 9 times of transition in the adaptive laboratory evolution in 10 g/L NaAc from MLB | This study |
| MLB12 | From the culture of 12 times of transition in the adaptive laboratory evolution in 10 g/L NaAc from MLB | This study |
| MLB14 | From the culture of 14 times of transition in the adaptive laboratory evolution in 10 g/L NaAc from MLB | This study |
| MLB16 | From the culture of 16 times of transition in the adaptive laboratory evolution in 10 g/L NaAc from MLB | This study |
| MLB17 | From the culture of 17 times of transition in the adaptive laboratory evolution in 10 g/L NaAc from MLB | This study |
| MLB18 | From the culture of 18 times of transition in the adaptive laboratory evolution in 20 g/L NaAc from MLB | This study |
| MLB21 | From the culture of 21 times of transition in the adaptive laboratory evolution in 20 g/L NaAc from MLB | This study |
| MLB24 | From the culture of 24 times of transition in the adaptive laboratory evolution in 20 g/L NaAc from MLB | This study |
| MLB27 | From the culture of 27 times of transition in the adaptive laboratory evolution in 20 g/L NaAc from MLB | This study |
| MLB30 | From the culture of 30 times of transition in the adaptive laboratory evolution in 20 g/L NaAc from MLB | This study |
| MLB33 | From the culture of 33 times of transition in the adaptive laboratory evolution in 20 g/L NaAc from MLB | This study |
| MLB36 | From the culture of 36 times of transition in the adaptive laboratory evolution in 20 g/L NaAc from MLB | This study |
| MLB40 | From the culture of 40 times of transition in the adaptive laboratory evolution in 20 g/L NaAc from MLB | This study |
| MLB43 | From the culture of 43 times of transition in the adaptive laboratory evolution in 20 g/L NaAc from MLB | This study |
| MLB46 | From the culture of 46 times of transition in the adaptive laboratory evolution in 20 g/L NaAc from MLB | This study |
| MLB46-05 | Single cloning separated from MLB46 | This study |

**Table S3 Full names and abbreviations of metabolites or genes**

| **Metabolites or Genes** | **Detailed description** |
| --- | --- |
| **Metabolites** |  |
| G-6-P | glucose-6-phosphate |
| F-6-P | fructose-6-phosphate |
| F-1,6-P | fructose-1,6-diphosphate |
| PEP | phosphoenolpyruvate |
| PYR | pyruvate |
| ACP | acetyl-phosphate |
| OAA | oxaloacetate |
| CIT | citrate |
| ICT | isocitrate |
| α-KG | α-ketoglutarate |
| Suc-CoA | succinyl-CoA |
| SUC | succinate |
| FUM | fumarate |
| MAL | malate |
| **Genes** |  |
| *glk* | glucokinase |
| *pgi* | glucose-6-phosphate isomerase |
| *fbp* | fructose-1,6-bisphosphatase I |
| *pykA* | pyruvate kinase II |
| *pykF* | pyruvate kinase I |
| *ppsA* | phosphoenolpyruvate synthetase |
| *pck* | phosphoenolpyruvate carboxykinase |
| *ppc* | phosphoenolpyruvate carboxylase |
| *aceA* | isocitrate lyase |
| *aceB* | malate synthase A |
| *aceE* | pyruvate dehydrogenase E1 component |
| *aceF* | pyruvate dehydrogenase, E2 subunit |
| *gltA* | citrate synthase |
| *acnAB* | aconitate hydratase |
| *icd* | isocitrate dehydrogenase |
| *sucAB* | α-ketoglutarate dehydrogenase |
| *sucCD* | succinyl-CoA synthetase |
| *lpd* | lipoamide dehydrogenase |
| *sdhABCD* | succinate dehydrogenase |
| *frdABCD* | fumarate reductase |
| *fumABC* | fumarase |
| *mdh* | malate dehydrogenase |
| *mqo* | malate dehydrogenase |
| *maeA* | NAD(+)-dependent malate dehydrogenase |
| *maeB* | NADP(+)-dependent malate dehydrogenase |
| *ackA* | acetate kinase |
| *pta* | phosphate acetyltransferase |
| *acs* | acetyl-CoA synthetase |
| *flgA* | flagellar basal body P-ring formation protein |
| *flgBCFG* | flagellar basal-body rod protein |
| *flgN* | flagellar biosynthesis protein |
| *flgKL* | flagellar hook-filament junction protein |
| *flgJ* | putative peptidoglycan hydrolase |
| *fliL* | flagellar protein |
| *fliI* | flagellum-specific ATP synthase |
| *fliMGN* | flagellar motor switch protein |
| *soxRS* | oxidative stress regulator |
| *rpoS* | stress responsive sigma factor |
| *cra* | global regulators |
| *iclR* | isocitrate lyase regulator |
| *fadR* | fatty acid degradation repressor |

**References**

1. Jiang M, Liu SW, Ma JF, Chen KQ, Yu L, Yue FF, Xu B and Wei P (2010) Effect of growth phase feeding strategies on succinate production by metabolically engineered *Escherichia coli.* *Applied and Environmental Microbiology* 76: 1298-300. <https://doi.org/10.1128/AEM.02190-09>
2. Liu Y, Wu H, Li Q, Tang X, Li Z and Ye Q (2011) Process development of succinic acid production by *Escherichia coli* NZN111 using acetate as an aerobic carbon source. *Enzyme and Microbial Technology* 49: 459-64. <https://doi.org/10.1016/j.enzmictec.2011.07.006>
3. Wang D, Li Q, Song Z, Zhou W, Su Z and Xing J (2011) High cell density fermentation via a metabolically engineered *Escherichia coli* for the enhanced production of succinic acid. *Journal of Chemical Technology & Biotechnology* 86: 512-8. <https://doi.org/10.1002/jctb.2543>
4. Wang J, Wang H, Yang L, Lv L, Zhang Z, Ren B, Dong L, Li N (2018) A novel riboregulator switch system of gene expression for enhanced microbial production of succinic acid. *Journal of Industrial Microbiology and Biotechnology* 45(4): 253-269. https://doi.org/10.1007/s10295-018-2019-3
5. Yu J-H, Zhu L-W, Xia S-T, Li H-M, Tang Y-L, Liang X-H, Chen T, Tang Y-J (2016) Combinatorial optimization of CO_2_ transport and fixation to improve succinate production by promoter engineering. *Biotechnology and Bioengineering* 113(7): 1531-1541. https://doi.org/10.1002/bit.25927
6. Yu Y, Zhu X, Xu H and Zhang X (2019) Construction of an energy-conserving glycerol utilization pathways for improving anaerobic succinate production in *Escherichia coli*. *Metabolic Engineering* 56: 181-9. <https://doi.org/10.1016/j.ymben.2019.10.002>
7. Zhang W, Zhu J, Zhu X, Song M, Zhang T, Xin F, Dong W, Ma J and Jiang M (2018) Expression of global regulator IrrE for improved succinate production under high salt stress by *Escherichia coli*. *Bioresource Technology* 254: 151-6. <https://doi.org/10.1016/j.biortech.2018.01.091>
8. Zhu L-W, Xia S-T, Wei L-N, Li H-M, Yuan Z-P and Tang Y-J (2016) Enhancing succinic acid biosynthesis in *Escherichia coli* by engineering its global transcription factor, catabolite repressor/activator (Cra). *Scientific Reports* 6: 36526. https://doi.org/10.1038/srep36526
