# Supplementary figures and images for: Effect of adaptive laboratory evolution of engineered Escherichia coli in acetate on the biosynthesis of succinic acid from glucose in two-stage cultivation
Source: Bioresour Bioprocess. 2024 Apr 5;11(1):34. doi: 10.1186/s40643-024-00749-5 (PMC10997558; doi:10.1186/s40643-024-00749-5)

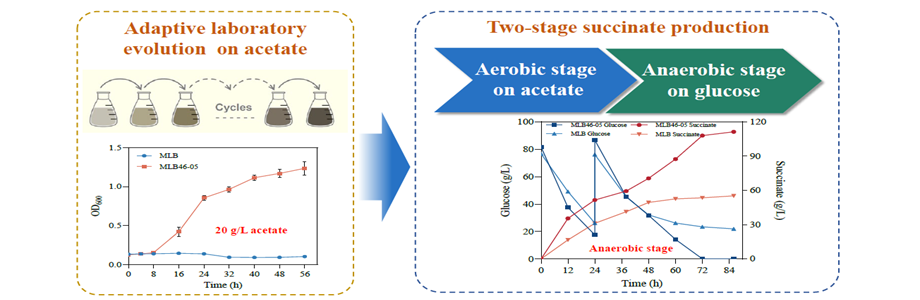

Supplement: Supplementary file 2 — Supplementary Material 2 [file 40643_2024_749_MOESM2_ESM.png]
